# The response to unfolded proteins in schizophrenia and bipolar disorder

**DOI:** 10.1192/j.eurpsy.2023.1324

**Published:** 2023-07-19

**Authors:** C. Cachán, I. M. Valle, Y. Potes, A. González Rubio, N. Menéndez Coto, D. López Fanjul, I. Vega Naredo, B. Caballero, P. Saiz, J. Bobes, P. García Portilla, A. Coto Montes

**Affiliations:** 1 University of Oviedo; 2 Servicio de Inmunología, Hospital Universitario Central de Asturias (HUCA); 3 Instituto de Investigación Sanitaria del Principado de Asturias (ISPA), Oviedo, Spain

## Abstract

**Introduction:**

Schizophrenia (SCH) and bipolar disorder (BD) are severe mental disordes, which have high incidence (Whiteford *et al.* Lancet 2013; 381 1575-86) and are the main causes of diasibility in young people (WHO 2022; https://www.who.int/news-room/fact-sheets/detail/mental-disorders).

Psycological stress appears in different mental disorders, and this is directly related to oxidative stress (Moller *et al.* Chem Biol Interact. 1996; 102 17-36)(Pupic-Bakrac *et al.* 2020; Psychiatr Danub. 32 412-9). Oxidative stress causes reticulum edoplasmic stress (ER stress) and this produces high levels of misfolded proteins. Defective proteins are degraded by the proteasome, but but when the density of misfolded proteins exceeds the capacity of the proteosome, the Unfolded and Misfolded Protein Response (UPR) is triggered through three main pathways: Inositol-requiring enzyme 1α (IRE1α); transcription factor 6 alpha (ATF6α) and protein kinase RNA-Like ER kinase (PERK), trying to recover normal protein synthesis capacity (Bermejo-Millo *et al.* 2018; Mol Neurobiol. 55 7973-86) (González-Blanco *et al.* 2022; J Cachexia Sarcopenia Muscle 13 919-31).

**Objectives:**

Characterizing ER stress and UPR in SCH and BD.

**Methods:**

We studied ER stress and UPR in peripheral blood mononuclear cells (PBMC) from 50 patients with SCH and an equal number of patients with BD compared to their corresponding controls in order to achieve our objectives.

Western Blot assay were performed following classical procedure () and the results was normalized to Ponceau as loanding control (Nie *et al.* 2017; BiochemByophys Resp 12 10-13) (Sander *et al.* 2019; Anal Biochem 575 44-53). Proteasome activity was assessed using Proteasome Activity Assay Kit (ab107921, Abcam, Cambridge, UK).

**Results:**

ER stress was evaluated with BiP/GRP78. Our results showed significantly increased expression in SCH (p<0,01) and BD (p<0,05), being more increased in SCH. Proteasome activity was increased in SCH and BD, being only statistically significant in SQZ (p<0,05). UPR study showed IRE1a cascade significantly activated in SCH (p<0,001) and only slight increased in BD showed without statistical differences. ATF6a pathway is measured by cleavage to active protein (50-kDa). Results showed higher expression in SCH than in BD and controls (p<0,001). In addition, PERK pathway showed higher statistical levels of p-eIF2a/eiF2a ratio in SCH than in BD and control respectively (p<0,05 and p<0,01).

**Conclusions:**

Our results showed a greater alteration in SCH than in BD at the level of protein synthesis, which implies a greater toxicity at the cellular level and, therefore, a clear risk for the survival of cells in this pathology.

**Disclosure of Interest:**

None Declared

